# Artificial intelligence and myocarditis—a systematic review of current applications

**DOI:** 10.1007/s10741-024-10431-9

**Published:** 2024-08-14

**Authors:** Paweł Marek Łajczak, Kamil Jóźwik

**Affiliations:** Zbigniew Religa Scientific Club at Biophysics Department, Faculty of Medical Sciences in Zabrze, Medical University of Silesia, Zabrze, Poland

**Keywords:** Artificial intelligence, Myocarditis, Deep learning, Machine learning, Automated diagnosis

## Abstract

Myocarditis, marked by heart muscle inflammation, poses significant clinical challenges. This study, guided by PRISMA guidelines, explores the expanding role of artificial intelligence (AI) in myocarditis, aiming to consolidate current knowledge and guide future research. Following PRISMA guidelines, a systematic review was conducted across PubMed, Cochrane Reviews, Scopus, Embase, and Web of Science databases. MeSH terms including artificial intelligence, deep learning, machine learning, myocarditis, and inflammatory cardiomyopathy were used. Inclusion criteria involved original articles utilizing AI for myocarditis, while exclusion criteria eliminated reviews, editorials, and non-AI-focused studies. The search yielded 616 articles, with 42 meeting inclusion criteria after screening. The identified articles, spanning diagnostic, survival prediction, and molecular analysis aspects, were analyzed in each subsection. Diagnostic studies showcased the versatility of AI algorithms, achieving high accuracies in myocarditis detection. Survival prediction models exhibited robust discriminatory power, particularly in emergency settings and pediatric populations. Molecular analyses demonstrated AI’s potential in deciphering complex immune interactions. This systematic review provides a comprehensive overview of AI applications in myocarditis, highlighting transformative potential in diagnostics, survival prediction, and molecular understanding. Collaborative efforts are crucial for overcoming limitations and realizing AI’s full potential in improving myocarditis care.

## Introduction

Myocarditis, a cardiovascular disease, is characterized by inflammation of the heart muscle. The causes include viral and bacterial infections, medication, toxins, and autoimmune disorders [1, 2]. The presence of viruses triggers an inflammatory response, leading to the release of molecules such as cytokines and perforins. Throughout this process, these molecules accumulate and inflict damage upon the myocardium [3–5]. Symptoms exhibited by individuals with myocarditis include chest pain, shortness of breath, palpitations, dull heart sounds, and dizziness. The progression of this disease may result in heart failure, cardiac arrest, stroke, heart attacks, or tachyarrhythmias [6]. Diagnostic methods such as ECG, histological examination (heart biopsy), troponin level, or cardiac MRI (CMR) are extensively employed. Treatment modalities vary based on etiology, clinical presentation, and disease stage, encompassing the use of immunomodulatory, immunosuppressive, and conventional therapies [7].

As the incidence of this life-threatening condition rapidly rises, accurate diagnosis becomes imperative for determining swift treatment strategies [8–10]. In response to this, rapidly advancing technology, such as artificial intelligence (AI), proves invaluable to physicians. Subsets of AI, mainly machine learning (ML) and deep learning (DL), demonstrate remarkable capabilities. DL algorithms, emulating human neurons, facilitate rapid multitasking, surpassing standard algorithms. The application of AI in medicine, particularly in cardiology, has gained traction, with notable uses in diagnosing myocardial infarction [11], differentiating heart-related conditions [12], risk prediction assessment [13], and electrophysiology, including the detection of arrhythmias [14].

Given the expanding potential and interest in incorporating artificial neuron models into cardiology, coupled with the escalating incidence of myocarditis, this paper seeks to consolidate current knowledge regarding AI applications in various aspects of myocarditis. Through a comprehensive review of AI applications, the aim is to address the current gap in the literature and offer insights for clinicians and researchers. We plan to analyze current applications of machine learning into diagnostics, survival and biomolecular analysis within myocarditis field, by analyzing the accuracy of the algorithms, effectiveness compared to routine methods, current limitations, future directions, and potential full-clinical integration.

The integration of AI technologies is poised to revolutionize myocarditis diagnosis, improve survival prediction accuracy, and unravel molecular complexities, thereby facilitating the implementation of tailored treatment strategies. This exploration aims to consolidate existing knowledge and pave the way for future research avenues in harnessing AI for more effective myocarditis management.

## Methodology

This work followed PRISMA guidelines for reporting systematic reviews [15]. To find articles for this synthesis, five databases were chosen: PubMed, Cochrane Reviews, Scopus, Embase, and Web of Science. MeSH terms such as artificial intelligence OR deep learning OR machine learning AND myocarditis OR inflammatory cardiomyopathy were used in search engine. Full search strategy is available in the Appendix to this article.

No filters were used in the databases; articles from all the years and languages were included. We have excluded non-original articles (reviews, editorials etc.), abstract-only works, articles utilizing conventional statistical methods, and studies focused on technical aspects and AI, rather than utilizing AI for myocarditis. Only original-type articles were included, with full text-available, which described use of AI in the myocarditis application. Two authors have independently screened articles, resolving conflicts on mutual agreement.

Additionally, a manual search for articles was performed independently by two authors on Google Scholar database. We have performed this, to capture articles, which might not have been indexed into the medical databases, as they could be indexed in electronic (engineering) journals.

From each study, we have extracted country of origin, aim of the study, model(s) of AI used in the work, validation technique, number of cases in the study (and myocarditis number if provided), source of data (imaging), and outcomes of the study. For diagnostic studies, we have also extracted modality used for the imaging, reference standard, and diagnostic accuracy values such as accuracy, sensitivity, *F*-score, precision, area under ROC curve (AUC), Dice score, or Hausdorff units.

## Results

**Fig. 1:**
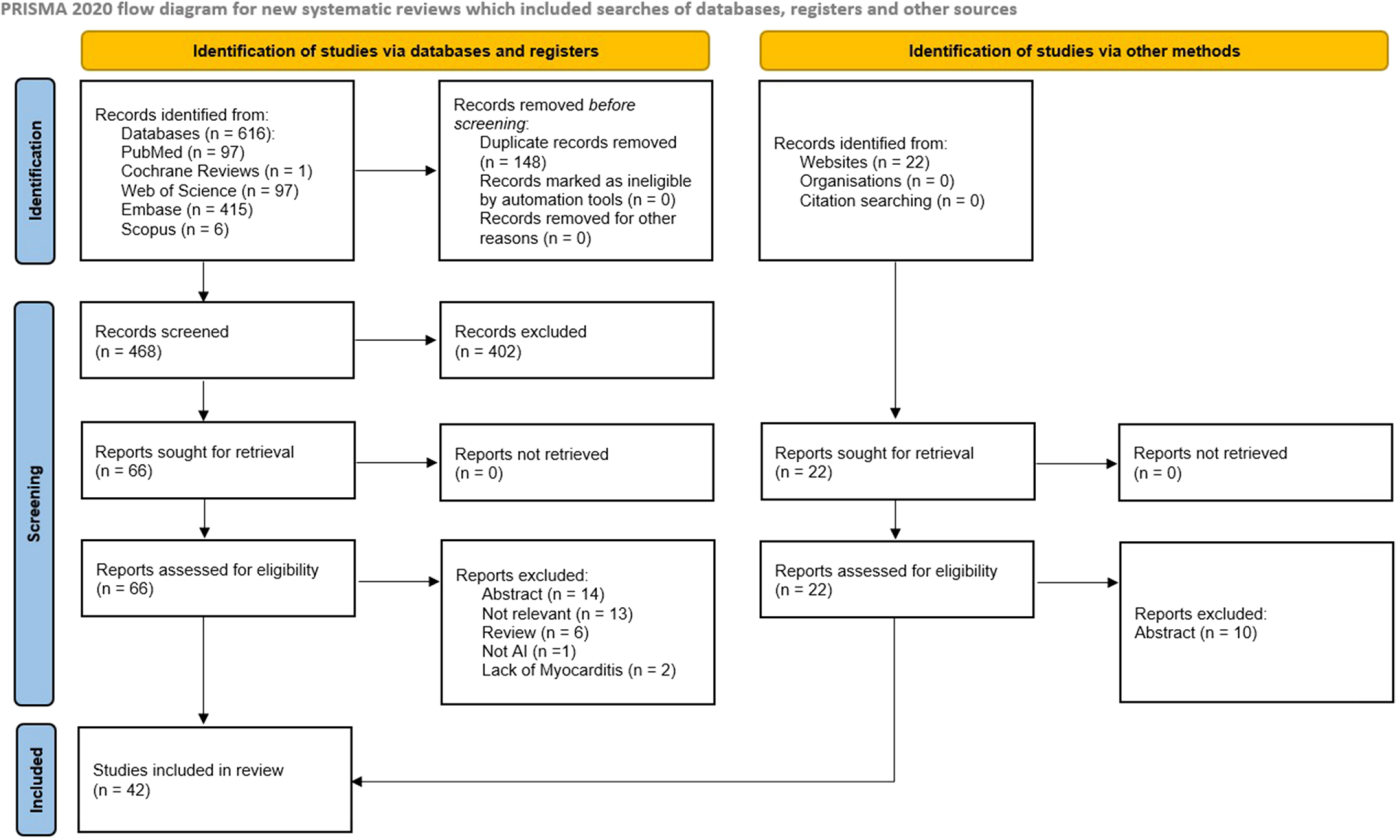
PRISMA flow diagram, made with PRISMA template [15]

On 25 December 2023, a search was performed for the relevant articles. From 5 databases, 97 works from PubMed, 1 from Cochrane Reviews, 97 from Web of Science, 415 from Embase, and 6 from Scopus. In total 616 articles had been found. Zotero software marked 148 duplicates for removal before the screening process. Two authors have independently screened 468 articles. After exclusion of 402 ineligible articles, 66 articles underwent the eligibility assessment. Finally, 30 articles from databases were included in this synthesis. Additionally, 12 articles were found from the manual search process, which fulfilled inclusion and exclusion criteria of this topic, and were included. A total of 42 articles were included in this synthesis [16–57]. Full search process is visualized on the PRISMA flow diagram (Fig. 1).

We have identified 32 diagnostic articles, 7 articles about survival, and 3 focused on molecular aspects.

Included studies are presented in tables under each subsection (Tables 1, 2, and 3), which contain country of origin, aim of study, type of AI used, validation, and the results.

- a) Diagnosis
  Early and differential diagnosis of myocarditis allows for faster preparation of treatment strategy, which can be especially beneficial for the patient, by reducing unnecessary procedures, such as coronary angiograms. Researchers are nowadays able to develop complex algorithms for such tasks. While endomyocardial biopsy (EMB) remains a gold standard for the diagnosis, the presence of false positive and negative results and sampling errors induces the need for alternative diagnostic tools. Non-invasive modalities are being applied into diagnosis, such as cardiovascular MRI (CMR). While CMR imaging can be helpful for the diagnosis, expertise within this modality is required to minimize interpretation bias; hence, AI tools might enhance the accuracy, minimize bias, and improve the general workflow for the physician.
  There are various subtypes of AI models; however, in this paper, the machine learning (ML) subfamily will be mostly discussed. Classic ML models use various mathematical and statistical approaches to solve the tasks. Among ML, we can distinguish supervised learning, unsupervised learning, and reinforcement learning [58]:
- Supervised learning focuses on training ML models on labeled data. Examples include linear regression, logistic regression, support vector machines (SVM), or neural networks
- Unsupervised learning models use unlabeled data (algorithm analyzes the data, tries to find suitable patterns for the desired output). Examples include clustering (i.e., *k*-means), anomaly detection, or t-Distributed Stochastic Neighbor Embedding.
- Reinforcement learning algorithms use reward/penalty system by interacting with an environment.
  Artificial neural networks (ANN), which belong to ML subfamily, are created to handle large amounts of the data for the analysis. These neural networks consist of many layers (just like human brain), to mimic function of biological neurons [59]. Among the ANN family, deep learning (DL) and convolutional neural networks (CNN) are widely used nowadays in the field of medicine. ANNs learn by adjusting the weights of the connections between neurons using algorithms such as backpropagation. This process involves calculating the error of the network’s output and propagating this error back through the network to update the weights. Neurons in ANNs use activation functions to determine whether they should be activated based on the weighted sum of their inputs.
  AI algorithms are able to perform these diagnostics tasks many times faster than human experts, while showing near-perfect accuracy compared to reference standard [28]. This allows to reduce time for the diagnosis, saving valuable time for treatment of the patient. Additionally, inter-observer variability is reduced, allowing for reproduction of the results.
  However, authors of the algorithms note that the accuracy of the AI models may be influenced by the quality of MRI scans (different protocols, image size, parameters of the scan, applied sequences, etc.), variability in the size, and number and location of the myocarditis lesions, which might vary between the patients [17]. Another limitation of the diagnostic studies is the reliance on single-center datasets. Multicenter datasets would minimize the overestimation of the results and improve the accuracy of the algorithms [21].
  Patients with acute myocarditis should be diagnosed with the shortest time possible, as they can suffer from severe dyspnea [30]. Differentiation of acute myocarditis with ML models and the use of CMR have already been proven to be faster and much more accurate than human experts [29, 30].
  Another aspect is the lack of eligibility for gadolinium contrast-enhanced CMR. Currently, tests are ongoing not only to perform diagnosis among patients with contraindications but also to reduce the potential comorbidities from the contrast injection and reduce associated costs [30].
  Other studies reporting analysis between MI and myocarditis showed good performance, reaching up to 90% accuracy [31].
  Reinforcement learning algorithms, which have been discussed earlier, are also being applied into the field of myocarditis. These RL models may address the issues of imbalance in datasets, which are quite common in medical imaging (i.e., number of myocarditis images is very limited, compared to disease-free patients) [32]. This leads to improved sensitivity and specificity for the myocarditis diagnosis, resulting in less case missed. The ability to continuously learn and improve leads to the enormous potential in the clinical practice, as these AI models can be adjusted to frequently changing cardiology guidelines.
  AI is also being applied into detection of elevated T2 segments, achieving high accuracy values; however, further evaluation is needed in this field [33].
  Major issue with CMR scans is the time-consuming aspect—long protocol and examination times might not be the best solution in case of the emergency, where delays in the examinations often occur, or there are insufficient slots for performing CMR [42]. ECG, which is another non-invasive technique, may also be used, as an alternative to CMR scanning, by providing higher detection rates of tachycardia, bradycardia, ST segment changes, or T wave changes with the use of neural networks, compared to traditional methods [34, 36]. As ECG diagnosis often requires expertise knowledge, due to the extensive variety and variability of the electrocardiograms, machine learning could revolutionize this field, enhancing accuracy, especially among inexperienced physicians, which have significantly lower knowledge than cardiologists [60]. This could also reduce fatigue and time needed for diagnosis [34].
  Wu et al. have applied backpropagation neural network (BPNN), which was optimized using the adaptive with differential evolution (ADE) algorithm for differentiation of MI, prior MI, myocarditis, and other abnormal ECG readings [23]. Their algorithm managed to achieve 99.5%, 99.0%, and 99.6% of sensitivity, specificity, and accuracy, respectively, in differentiation between normal, abnormal, myocarditis, myocardial infarction, and prior myocardial infarction in a validation set comprising 1040 ECG signals. In external testing sets, the algorithm not only showed excellent accuracy (99.4% in ClinicalTrials.gov database and 99.6% in ICIs-myocarditis database set), which consisted of over 400 patients, but also showed superiority to SVM, RF, and even CNN algorithms.
  Acute myocarditis requires precise and fast differentiation from other cardiovascular conditions, during the patient admission at the emergency department. While CMR is a powerful tool for the diagnosis of the cardiac pathologies, this type of imaging exam often results in the delay between admission and diagnosis of the lesion. Rahman et al. have proposed an alternative ML algorithm to differentiate myocardial infarction and myocarditis based on clinical data only from patients presenting chest pain [21]. This data included troponin level, age, tobacco, sex, FEVG, diabetes, and overweight—first three parameters weighted more, due to their importance for detection for the algorithm. Several ML classifiers were included such as support vector machine (SVM), K-nearest neighbors (KNN), RF, extremely randomized tree (ERT), and more. These classifiers were coupled with unbalancing techniques such as Stratified, Under-sampling, Oversampling, or NearMiss. Algorithms were validated with tenfold cross-technique and tested on 537 cases. Oversampling technique with stacking gained the highest recall of 96.7% among all models for detection of myocarditis. It is worth noting that most of the algorithms (except stacking) required less than 5 s to execute the training and pre-validation process with over 300 images, showing savings not only in time but also in the resources, as non-expensive hardware was used in this process (regular laptop with integrated GPU).
  Zaman et al. have developed a natural language processing for analysis of text-based CMR reports, which included normal, dilated cardiomyopathy, hypertrophic cardiomyopathy, myocardial infarction, and myocarditis cases [22]. On a testing set with 400 cases, the algorithm revealed a sensitivity of 89% for myocarditis case detection. Moreover, this model required only 0.2 s to annotate over 1000 CMR reports. Notably, a classic SVM model showed comparable F1 scores in all pathologies, except myocarditis—authors note that the features of myocarditis overlap with other conditions, making the diagnosis more challenging.
  Other non-invasive modalities include cardiac echo, which is another solution and alternative for poor-quality CMR images [47]. Algorithms can be used for reproductive and highly accurate LVEF measurements, minimizing the operator subjectivity during the assessment, improving the decision-making, as AI can provide less over-estimated results of the LVEF. Again, assistance of AI can highly benefit novices to provide more accurate LVEF results [61, 62].
  Artificial intelligence has also been applied into the field of segmentation. Cardiac MRI may be used as a diagnostic tool for ejection fraction (EF) and ventricular volume measurements. LV EF is a major prognostic factor in acute myocarditis and heart failure [63, 64]. Because this process requires very high accuracy, an AI tool could improve the efficacy of this process. Manual segmentation performed by a physician not only is prone to intra-observer variability between specialists but also requires a lot of time, making this process from an economical perspective ineffective [65–67]. Additionally, the borders of the ventricle may vary due to the anatomical and lesion variations. This is even more complicated in case of the segmentation of the right ventricle compared to the left ventricle, due to the irregular, trabeculated walls [68]. AI could not only speed up this but also reduce the potential bias of the segmentation, making the diagnosis and follow-ups more effective [16, 20, 33, 39].
  AI has been also applied into segmentation of the ventricles, labeling CMR reports, functional analysis of ventricles, and more with myocarditis diagnosis (Table 1).
- b) Survival and risk prediction
  Prognosis and the survival prediction is invaluable tool for the further treatment steps. Baritussio et al. have developed an RF algorithm for survival predictor analysis in case of low number of events at follow-up [48]. In case of low number of events at the follow-up, the traditional multivariable Cox proportional hazards model may not be recommended, and machine learning models are more appropriate [69]. Random forest algorithm addresses the shortcomings of Cox model, in cases where the event of interest is infrequent. It excels at detecting intricate relationships between outcomes and covariates, even when dealing with numerous predictors and a few events [70–72]. They have implemented LV ejection fraction measure, positive antinuclear autoantibodies, biopsy proven status, C-reactive protein (CPR) level (which analysis was main goal of the study), clinical, imaging, and more laboratory data into ML model. CRP has been widely associated with cardiovascular diseases [73, 74]. One paper suggested that its elevated level may predict inflammatory cardiomyopathy [75]. The model achieved AUC of 0.907 for 369 patients in the testing set. The study found limited value of CRP level into the event prognosis.
  Chou et al. have assessed pediatric myocarditis mortality prediction with AI on a retrospective dataset [49]. The study compared traditional linear regression model to ML RF algorithm. Fourteen variables such as mechanical ventilation, cardiac arrest, acute kidney injury, or ventricular fibrillation were used to prognoses the mortality. Testing set comprised 1449 of patients—conventional linear regression achieved higher accuracy (95% vs 86%), however much lower sensitivity (53% vs 90%) compared to ML model in the testing set, showing random forest superiority to traditional statistical methods.
  Chou et al. have used the national Kids’ Inpatient Database, and a machine learning algorithm (RF), to discern associations between specific comorbidities, procedural interventions, and adverse outcomes in pediatric myocarditis [51]. The random forests’ algorithm was implemented for mortality factors prediction. The algorithm’s capacity to handle categorical variables and tolerate collinearity issues made it particularly suited for this analysis. Chou identified several key prognostic factors independently associated with increased mortality and prolonged hospital stay in pediatric myocarditis. Noteworthy contributors included comorbidities such as brain injury, acute kidney injury, dysrhythmias, coagulopathy, sepsis, and the utilization of extracorporeal membrane oxygenation (ECMO).
  Machine learning allows for better prediction of outcomes in survival tasks, by analyzing complex interactions between various risk factors. Even with limited number of variables, like in real clinical scenario, ML algorithms are able to accurately prognoses events among the diseased patients, with just as low as 20 variables [50]. This lays a groundwork for future prospective studies aiming at risk stratification and personalized management strategies in the realm of pediatric myocarditis, as ML solves the problem of small cohort studies—algorithms can be applied into large datasets to quickly find the potential outcome-associated risk factors in myocarditis [51, 76–78]. However, more testing is needed to resolve the heterogeneity between accurate identification of various biomarkers and risk factors [52].
- c) Biomolecular analysis and other applications
  Stefanovic et al. have explored the application of neural network models in understanding and predicting immune checkpoint inhibitor (ICI)–related myocarditis [55]. Due to the limited frequency of the ICI-related myocarditis, development of a clinical disease model would be a challenging task. Additionally, limited data collected and lack of time-related clinical signals from the patients created even more problems. Traditional machine learning methods face challenges in analyzing time-related changes and magnitudes in individuals due to limited data and sparse time-related clinical signals. Authors have employed an alternative approach, utilizing NN models on patient medical records enriched for ICI-related myocarditis cases. Their model incorporated known clinical covariates and structured scoring systems, providing insights into the time and magnitude of ICI-related myocarditis development, severity, treatment response, and resolution. Their research involved a longitudinal cohort of patients treated with ICIs, focusing on those with elevated serum troponin concentrations. Data, collected from electronic health records, included outpatient and inpatient healthcare claims, clinical monitoring, laboratory results, and pharmacy data. The NN model outputs parameters such as myocarditis severity score, troponin levels, and composite parameters related to tumor burden, combination immune-related adverse events (irAEs), lab signals, and myocarditis signs and symptoms. A total of 15 cases of ICI-related myocarditis were identified, leveraging rich time series clinical data to construct an NN-based predictive model. The model analyzed determinants of myocarditis onset, severity, response to treatments, and resolution. Key predictors included combined myocarditis metric, combined lab signal, CPK-MB, ALT, troponin, and QRS interval. NN modeling allows for handling large amounts of time and magnitude-related data elements from electronic health records. Such approach enables an understanding of ICI-related myocarditis, including early detection, severity, treatment response, and outcomes.
  Li et al. have analyzed the molecular mechanisms of myocarditis through the exploration of miRNA networks, with the use of artificial intelligence, as they can be used as biomarker for various diseases [56, 79]. AI was used in processing and interpreting large-scale datasets from repositories—Gene Expression Omnibus (GEO). The study included data from GSE126677 and GSE4172 databases, which were used to identify specific miRNA and mRNA expression patterns in myocarditis. The differential expression analysis revealed a considerable number of upregulated and downregulated miRNAs and mRNAs. The construction of a PPI network unveiled 13 core genes that play crucial roles in the context of myocarditis. These core genes could serve as potential biomarkers or therapeutic targets, offering future research. The miRNA-mRNA regulatory network analysis analyzed interactions between miRNAs and their target genes, observed in myocarditis. The study’s findings suggest that specific miRNAs, such as miR-21, miR-146b, miR-155, and miR-148a, may be implicated in the inflammatory responses associated with viral myocarditis, what has been also reported in the literature [80–86]. The identified miRNA-mRNA interactions, core genes, and enriched pathways contribute to a deeper understanding of myocarditis pathogenesis and offer a background for future investigations.

**Table 1:** Diagnostic applications of AI into myocarditis

Study	Aim	Modality	AI	Source of data	Dataset size (myocarditis cases)	Validation; training-validation-testing	Gold standard	Dice; Hausdorff	Sensitivity; specificity	Accuracy; *F*-score; other metric	Precision; AUC
Lou [16], China 2021	Segmentation of RV	CMR	Cascaded FC-DenseNet with level set method (Dense Convolutional Networks)	North China Theater General Hospital	45 (N/A)	Split, 15:0:30	One clinician	0.87 ± 0.11; 7.55 ± 7.25	N/A	N/A	N/A
Zhou [17], China 2023	Myocarditis detection	CMR	CNN with decision layer, generative adversarial network (GAN) algorithm, an enhanced differential evolution (DE), and a reinforcement learning for training	Z-Alizadeh Sani myocarditis dataset	N/A	Fivefold cross	N/A	N/A	88.6 ± 2.8; N/A	90.8 ± 2.0; 87.8 ± 2.5	87.1 ± 4.0; N/A
Kanjee [18], USA 2023	Diagnosis of complex conditions through case report study	Conference Paper Text	GPT-4 Chat	NEJM clinicopathologic conference papers	70 (N/A)	N/A	Three clinicians	N/A	N/A	Correct diagnosis in 45/70 cases (64%)	N/A
Attia [19], USA 2020	Detection of LV dysfunction	ECG	CNN Keras framework	Mayo Clinic	27 (1)	N/A	N/A	N/A	N/A	N/A	N/A; 0.95
Barbaroux [20], UK 2023	Segmentation of LV	CMR	NnU-Net—one 2D, second 2D with time network	Royal Brompton Hospital, King’s College London	260 (36)	Fourfold cross, 80%:0%:20%	N/A	0.84 ± 0.04; 3.8 ± 1.0	86 ± 7; N/A	N/A	83 ± 8; N/A
Rahman [21], France 2023	Myocarditis detection	Clinical Data, DE-MRI (reference)	ML—support vector machine (SVM) classifier, K-nearest neighbors (KNN), random forest (RF), extremely randomized tree (ERT), gradient boosting (GB), decision tree (DT), eXtreme gradient boost (XGB), light gradient boost machine (LGBM), and stacked generalization (stacking); DL—multilayer perceptron (MLP)	EMIDEC challenge, University Hospital of Dijon	537 (179)	Tenfold cross	N/A	N/A	96.65 (stacking OS); N/A	N/A; 97.46	98.3; N/A
Zaman [22], UK 2021	Labeling CMR from clinical radiology reports	Text CMR reports	Bidirectional encoder representations from transformers (BERT)	Three London hospitals	1503 (29)	Split, 801:302:400	Two clinicians with CMR experience	N/A	89; N/A	N/A; 86	83; 0.97 (95% CI: 0.92, 1.00)
Wu [23], China 2023	Myocarditis detection	ECG	Improved quantum genetic algorithm (IQGA), adaptive differential evolution optimization backpropagation neural network (BPNN)	Chinese PLA General Hospital (Internal); ClinicalTrials.gov, ICIs-myocarditis database (External)	Internal 5200 (1200); external 466 (466)	Fivefold cross, external	Two cardiologists	N/A	99.45; 98.95 (results for internal set)	99.59; 97.93	N/A
Sharifazi [24]; Multinational 2022	Myocarditis detection	CMR	CNN with *k*-means clustering	OMID hospital, Iran	98,898 (61,334)	Tenfold cross	N/A	N/A	95.7; 98.56 (4 cluster model)	97.41; 96.5	97.6; 0.9705
Borodyansky [25], Russia 2022	Detection of thoracic diseases	X-ray	Inceptionv3	Kaggle chest X-ray images	N/A	N/A	N/A	N/A	N/A	74; N/A	N/A
Masutani [26], USA 2023	Detection of wall motion abnormalities	CMR	Modified U-Net	Institution	219 (N/A)	Split	Four board-certified cardiothoracic radiologists	N/A	86 (95% CI, 85, 87); 85 (95% CI, 79, 91)	86 (95% CI, 85, 87); N/A	N/A; 0.9
Yuan [27], China 2019	Evaluation of early gadolinium enhancement (EGE) and left ventricular functional parameters	CMR	Not specified AI model	Chengdu Medical College	21 (21)	N/A	Two operators	N/A	N/A	N/A	N/A
Böttcher [28], Germany 2020	Quantification and function analysis of LV	CMR	Cvi42	Institution	50 (11)	N/A	Board-certified cardiac radiologist	N/A	N/A	N/A	N/A
Cau [29], Italy 2022	Diagnosis of takotsubo cardiomyopathy		RF, extremely randomized trees, plain bagging classifier of decision trees, adaptive boosting, extreme gradient boosting	Institution	43 (14)	Nested leave-one-out cross	Cardiovascular radiologist	N/A	91.6 (95% CI, 78, 100); 85.8 (extremely randomized trees)	N/A	N/A; 0.94 (95% CI, 0.90, 0.99)
Cavallo [30], Italy 2022	Radiomics prediction of late gadolinium enhancement in patients with acute myocarditis	CMR	RF, Neural network (Nnet), Naïve Bayes (NB), XGB, SVM with radial basis function kernel (svmRadial), SVM with linear kernel (svmLinear), knn, partial least squares (pls), decision tree (CART), logistic regression model (glm), linear discriminant analysis (lda)	Policlinico Tor Vergata, Italy	19 (19)	Tenfold cross	Two radiologists	N/A	80; 73 (ensemble model)	75; N/A	N/A; 0.79 (95% CI, 0.66, 0.92)
Di Noto [31], Switzerland 2019	Diagnosis of myocarditis from MI	CMR	Linear discriminant analysis (LDA), k-nearest neighbor (k-NN), multilayer perceptron, SVM, TreeBagger (TB)	Institution	173 (62)	Tenfold cross, 140:16:17	Cardiovascular imaging expert	N/A	94 ± 1; 80 ± 2 (LDA 3D analysis)	89 ± 1; N/A	89 ± 2; N/A
Moravvej [32], Iran 2022	Myocarditis detection	CMR	Reinforcement learning with population-based algorithm	Omid Hospital, Iran	7135 (4686)	Fivefold cross	N/A	N/A	86.3 ± 1.7; 90.1 ± 2.4	88.6 ± 2; 85.1 ± 2.4	N/A
Kim [33], Korea 2023	Segmentation of LV myocardium, identification of elevated T2 values	CMR	Myomics	Dongsan and Severance hospitals	83 (41)	N/A	Two cardiac radiologists	0.848 ± 0.085 (segmentation)	83.6–92.8; 82.5–92.0 (detection for all cases)	82.7–92.2; N/A	N/A
Li [34], China 2020	Detection of pediatric myocarditis	ECG	ANN	Physio Bank Database	74 (74)	N/A	N/A	N/A	N/A	Positive detection rate 97.4%	N/A
Ulloa [35], Spain 2023	Myocardial feature tracking (FT) and DL strain (DLS) analysis in acute myocarditis	CMR	Cvi42	Institution	37 (17)	N/A	Cardiologist and radiologist	N/A	N/A	High inter/intra-observer variability of 0.97–1.00	N/A
Cuadros [36], Chile 2019	QT interval estimation	ECG	SVM	PhysioNet	97 (4)	External	N/A	N/A	N/A	Error 3.11% ± 2.48	N/A
Ghareeb [37], Qatar 2022	Pattern analysis in acute myocarditis	CMR	K-means with Bayesian factor analysis	Heart Hospital, Qatar	169 (169)	N/A	N/A	N/A	N/A	Identification of two inflammation patterns	N/A
Joy [38], UK 2021	LV function analysis after SARS-CoV-2	CMR	CNN	COVIDsortium patients from 3 London hospitals	149 (4%)	N/A	One expert	N/A	N/A	N/A	N/A
Overhoff [39]. Germany 2021	Myocardial segmentation	CMR	U-Net (2D)	Six medical universities	47 (47)	Split, 80%:10%:10%	Cardiac radiologist	0.813 (0.941 for blood pool)	N/A	N/A	N/A
Kasmaee [40], Iran 2024	Myocarditis detection	CMR	ELRL-MD	Z-Alizadeh Sani myocarditis dataset	7135 (4686)	fivefold cross	N/A	N/A	88.8 ± 1.8; 92.5 ± 2.5	91.1 ± 1.9; 88.2 ± 2.3	87.7 ± 3.8; N/A
Ribeiro [41], Portugal 2024	Diagnosis of myocarditis from 8 CVDs	ECG	19 ML classifiers	PTB diagnostic ECG database	483 (3)	Leave-one-out cross	N/A	N/A	72.93; N/A	81.16; 76.34	81.16; 0.5552
Paciorek [42], Germany 2024	Diagnosis of myocarditis from CVDs	CMR	DenseNet-161	German Heart Center Munich	200 (36)	Split, 65%:15%:20%	Two CMR experts	N/A	100; 38	88; N/A	N/A; 0.75
Yang [43], Malaysia 2024	Myocarditis detection	CMR	Deep reinforcement learning and an improved differential evolution algorithm	Z-Alizadeh Sani myocarditis dataset	7135 (4686)	Fivefold cross	N/A	N/A	88.9 ± 3.8; 92.6 ± 2.1	91.0 ± 1.9; 88.3 ± 2.9	87.5 ± 4.3; N/A
Danaei [44], Iran 2022	Myocarditis detection	CMR	ML-Artificial Bee Colony	Z-Alizadeh Sani myocarditis dataset	893 (586)	Fivefold cross	N/A	N/A	87.1 ± 2.2; 91.0 ± 2.5	89.5 ± 1.8; 86.2 ± 2.2	85.4 ± 3.6; N/A
Wang [45], China 2024	Detection of CVDs	CMR	DL Video-based Swin transformer	Multiple PRC hospitals	Internal 6650, external 1416 (total 156 myocarditis)	Threefold cross, external	Group of CMR experts	N/A	96.6; 97.1 (internal)	N/A; 72.4	N/A; 0.987
									89.8 ± 1.8; 93.1 ± 3.2 (external)	91.2 ± 3.0; 86.2 ± 3.2	88.3 ± 5.1; N/A
Golilarz [46], Iran 2024	Myocarditis detection	CMR	CNN with generative adversarial network-based data augmentation	Z-Alizadeh Sani myocarditis dataset	7135 (4686)	Fivefold cross	N/A	N/A		91.2 ± 3.0	
Sveric [47], Germany 2024	Assessment of LV EF	Echocardiography	LVivo Seamless™	Herzzentrum Dresden, Technische Universität Dresden, Germany	301 (48)	N/A	Three cardiologists	N/A	78; 92	N/A	N/A

**Table 2:** Survival analysis applications of AI in myocarditis

Study	Aim	AI	Source of data	Dataset size (myocarditis cases)	Validation	*Event prediction*	*AUC; accuracy*	Sensitivity; specificity
Baritussio [48], Italy 2022	Analysis of C-reactive protein	RF	Department of Cardiac, Thoracic, Vascular Sciences and Public Health, University of Padua, Italy	369 (369)	Internal	3 years	0.903 (95% CI 0.837, 0.968); N/A	N/A
Chou [49], China 2021	Prediction of pediatric myocarditis	RF	Kids’ Inpatient Database	4144 (4144)	Fivefold cross, 65%:0%:35%	N/A	0.94; 86	89.9; 85.8
Heilbroner [50], USA 2021	Predicting cardiac adverse events in patients receiving immune checkpoint inhibitors	XGBoosted decision tree	CancerLinQ database (American Society of Clinical Oncology)	4960 (1)	80%:0%:20%	20, 40, 60, 80, 100, 120, and 140 days	0.65 (95% CI: 0.58, 0.75) at 100 days; N/A	N/A
Chou [51], China 2021	Identification of prognostic factors for pediatric myocarditis	RF	Kids’ Inpatient Database	7241 (7241)	tenfold cross	N/A	N/A	N/A
Li [52], China 2024	Predicting myocarditis with COVID-19 patients	RF	Guangzhou Medical University and Dazhou Central Hospital	5230 (562)	Split, 70%:0%:30%	N/A	0.887; N/A	77.6; 91.7
Nogimori [53], Japan 2024	Prediction of pediatric adverse cardiovascular events from ECG	CNN	University of Tokyo Hospital	1180 (11)	Split, 85%:12.5%:12.5%	180 days	0.826 (95% CI: 0.706, 0.945); 66.5	87.5; 65.5
Stephens [54], Australia 2023	Survival prediction in venoarterial extracorporeal membrane oxygenation	Deep neural network	Extracorporeal Life Support Organization (ELSO) registry (Internal); ELSO database (External)	Internal 18,167 (397), external 5015 (65)	Fivefold cross, external	N/A	0.8; 69.2 (external)	73.2; N/A

**Table 3:** Biomolecular analysis and other applications

Study	Aim	AI	Source of data	Dataset size (myocarditis cases)	Validation	Outcome
Stefanovic [55], USA 2022	Modeling immune checkpoint inhibitor myocarditis	ANN	Roswell Park Comprehensive Cancer center	23 (15)	Leave-one-out	Various heterogeneity reported in the error prediction
Li [56], China 2022	Connectivity and regulatory mechanisms of miRNA-mRNA networks in myocarditis	DL	GEO database	15 (10)	N/A	See main text
Brunner [57], Austria 2022	Quantification and analysis of inflammatory cells (CD3 and CD45 positive cells) in myocarditis	ANN	Institute of Forensic Medicine, Medical University of Innsbruck	40 (38)	N/A	Successful separation between the positively stained cells for each marker, the negative stained nuclei, and the background composed of muscle fibers and vessel-bearing supporting tissue

## Discussion

This systematic review brings forth the transformational role of artificial intelligence in the diagnosis, prognosis, and molecular analysis of myocarditis. The use of AI in general and the specific integration of it into healthcare, particularly in the context of myocarditis, gives a turning point from a traditional approach to the diagnostic and therapeutic aspects. To the best of our knowledge, this is the very first systematic review in the literature concerning this subject.

### Diagnostic applications

AI has thus been particularly effective in improving the accuracy and efficiency of imaging modalities such as cardiovascular MRI (CMR), electrocardiograms (ECG), echocardiography, or even text reports for myocarditis diagnostics. For example, AI algorithms involving deep learning models and convolutional neural networks have demonstrated high-level accuracies in myocarditis detection over humans, thus giving potential value in shortening diagnostic time and enhancing greater chances of successful outcomes due to early treatment.

Furthermore, research has found AI to help distinguish myocarditis from other cardiac pathologies. It is therefore often able to overcome the challenge presented by such subtle presentation of myocarditis, overlapping with most other cardiac conditions.

Other applications include the segmentation of the ventricles in myocarditis, which are quite challenging tasks, due to the time-consuming nature, as well as potential operator bias. AI has been validated in this field, reaching high Dice (segmentation) scores, in relatively short amount of time compared to the human experts.

Application in other non-invasive modalities, such as echocardiography or ECG, while being less present in this systematic review compared to CMR, brings potential for even faster diagnosis, especially in the queued ICU and radiology units, or in places where there is no access to CMR. This shows that the integration of the AI into the clinical practice for myocarditis does not need to be limited to tertiary care, as ECG systems are nowadays present in most primary care units.

The potential application of AI in diagnostics is hampered by imaging protocol variations and scan quality. Furthermore, single-center data characterizes a lot of diagnostic models, which ends up limiting their generalizability. Generalization may be enhanced by developing AI tools from multicenter datasets with strong robustness and accuracy of AI diagnostics.

### Prognostic and survival predictions

AI models have also assisted in predicting patient outcomes, particularly in pediatric cases and those with complex clinical presentations. The use of machine learning algorithms, including random forests, has been important in predicting long-term survival and immediate clinical requirements for patients. Such AI-driven prognostic tools are promising sources of revealing intricate relationships between clinical parameters and patient outcomes that help guide critical treatment decisions and resource allocation.

For example, AI has been used to significant effect in determining survival probabilities for patients with myocarditis, using numerous clinical inputs to calculate outcomes with greater precision than conventional statistical models, such as multivariable Cox hazards model. This has particular implications for settings with low event rates, where traditional models tend to do poorly.

### Molecular insights and immunological applications

Beyond diagnosis and prognosis, AI is heading the understanding of molecular and immunological aspects of myocarditis. AI has been used to recognize biomarkers and genetic factors related to myocarditis by analyzing vast datasets. It has served purposes in understanding its pathogenesis and, hence, potential therapeutic targets. Immense help in developing a framework for the prediction of immune checkpoint inhibitor-related myocarditis onset and severity—critical for tailoring individualized treatment plans—has been done using machine learning applied to the analysis of immune checkpoint inhibitor-related myocarditis.

### Future directions and challenges

Future applications in AI for myocarditis care will benefit from increased scaling in datasets across all populations, enhanced interpretability and transparency of the models, and overcoming current regulatory and ethical issues surrounding AI deployment in clinical settings through the joint collaborative efforts of clinicians, researchers, and technologists.

In addition, continuous research efforts should, therefore, concentrate on developing increasingly improved AI models regarding the handling of the diversity and variability within clinical data and an increased level of adaptiveness to new information and clinical guidelines. Hence, to operationalize the benefit that AI could bring to the management of myocarditis, there will be a need to make AI tools accessible to healthcare professionals and to fit these tools into existing healthcare infrastructures.

### Limitations

Major limitation of some of the included studies is the small amount of the myocarditis cases—some of the studies have not reported detailed number of myocarditis, but mentioned only use of such cases or myocarditis cases accounted for less than 1% of dataset. While these studies show promising overall results, this prevents from making definite conclusions in case of myocarditis and limits potential clinical application of AI technology. On top of that, researches often included data from only one institution, which also limits generalizability in case of different populations. External validation is the foundation of the proper validation of the AI in the real-world scenario. Multiple institutions or more internet-available datasets should be used during the model testing, as myocarditis lesion often vary between the patients in terms of size. Retrospective study design with unclear exclusion criteria only increases bias within the results.

Additionally, some methodologies were reported in very few (or even one) studies (for example, echocardiography); hence, full evaluation of the application of the artificial intelligence remains very limited. More studies should cover these modalities.

We have also encountered enormous disproportion between the number of diagnostic, survival, and molecular analysis studies—literature reported three times more diagnostic studies (32), than others combined (10). This aspect should also be covered in the future, as data related to the survival analysis and event prognosis remains relatively scarce.

Lack of randomized controlled trials, very few (and relatively limited) evaluations to human expert studies, and small number of prospective studies should be taken into consideration during the planning of future trials, to fully integrate AI into healthcare units for myocarditis diagnosis.

Finally, we have not encountered any major cost-effectiveness analysis for the AI application. The economical perspective analysis is crucial for implementation into hospital settings.

## Conclusion

AI technologies hold great potential as they progress across the domains of diagnostics, prognostics, and understanding the molecular dynamics of the disease. As AI progresses, the integration of such towards myocarditis management changes towards greater personalization, efficiency, and effectiveness. However, success in such efforts will depend on a multidisciplinary approach spanning technology and medicine that can foster innovation that is both technically feasible and clinically valuable, all within an ethically sound ambit.

## Data Availability

All data collected during the synthesis was presented within the manuscript and/or tables. Authors declare no additional data available.

## Appendix Search strategy

PubMed – (deep learning OR convolutional neural network OR computer-aided learning OR machine learning OR artificial intelligence) AND (myocarditis OR (inflammatory cardiomyopathy)).

Cochrane – (deep learning OR convolutional neural network OR computer-aided learning OR machine learning OR artificial intelligence) AND (myocarditis OR (inflammatory cardiomyopathy)) in Title Abstract Keyword.

Web of science – (deep learning OR convolutional neural network OR machine learning OR artificial intelligence) AND (myocarditis OR (inflammatory cardiomyopathy)).

Embase – (“deep learning”/exp OR “deep learning” OR (deep AND (“learning”/exp OR learning)) OR “convolutional neural network”/exp OR “convolutional neural network” OR (convolutional AND neural AND (“network”/exp OR network)) OR “computer-aided learning” OR (“computer aided” AND (“learning”/exp OR learning)) OR “machine learning”/exp OR “machine learning” OR ((“machine”/exp OR machine) AND (“learning”/exp OR learning)) OR “artificial intelligence”/exp OR “artificial intelligence” OR (artificial AND (“intelligence”/exp OR intelligence))) AND (“myocarditis”/exp OR myocarditis OR “inflammatory cardiomyopathy”/exp OR “inflammatory cardiomyopathy” OR (inflammatory AND (“cardiomyopathy”/exp OR cardiomyopathy))).

Scopus – (deep AND learning OR convolution AND neural AND network OR computer-aided AND learning OR machine AND learning OR artificial AND intelligence) AND (myocarditis OR (inflammatory AND cardiomyopathy)).
